# Immunometabolism and inflammation: a perspective on animal productivity

**DOI:** 10.1093/af/vfac060

**Published:** 2022-10-14

**Authors:** Ellen Davis

**Affiliations:** Arm & Hammer Animal and Food Production, Princeton, NJ, USA

**Keywords:** homeostasis, immune development, proinflammatory

**Implications**

- Immunometabolism is the basis for allocating nutrient resources between growth and survival in animal production.
- Inflammation is the cost of animal productivity and represents a mitigation point for managing the nutrient resources between growth and survival.
- Research gaps related to how early life events influence immunometabolism and metabolic programming throughout an animal’s productive life should be further investigated.

It is well known that the inflammatory process has a profound impact on growth and metabolism. As early as the late 1990’s, Spurlock (1997) recognized that immune system activation in response to a pathogenic challenge occurred at the expense of energy resources for growth and productivity in livestock and poultry. This interface between immunology and metabolism has been further investigated, both at the cellular level related to the nutrient partitioning to support immune cell activation and the systemic level related to the partitioning of nutrients for tissue growth or turnover. Knowledge in the field of immunometabolism has made significant progress over the past 25 years with the development of investigative tools in genomics and molecular biology, such that there is a good mechanistic understanding at the cellular and tissue level of how immunity influences metabolic processes. Most lacking is an understanding of the intrinsic and external signals that instigate the immune response and the resulting metabolic changes influencing tissue repartitioning. Much of the scientific investigation of the interface between the immune system and metabolic function has been conducted within the human medical field to better understand diseases like obesity and cancer (Lercher et al., 2020) but many of the concepts can be used to inspire research direction to better understand how health and productivity of agricultural animals can be optimized.

## Immunometabolism and Host Homeostasis

The prevailing thought is that the function of the immune system is to act as a defense system against pathogens and “non-self” environmental challenges. At a conference presentation over a decade ago, Dr. Gary Huffnagle (Department of Internal Medicine at the University of Michigan) defined the purpose and function of the immune system, not as a host defense system, but as a mechanism to maintain host homeostasis, or an internal steady state. Although this concept that the immune system functions in homeostatic control and not as host defense from pathogens is unconventional, conceptually every immune system response is a reaction to some disruption of homeostasis. Regardless of whether that disruption is an invading bacterial pathogen or a physiological stress response, immune system activity is a reaction to a perturbation in the host’s physiological steady state and it functions to restore homeostasis.

Organisms closely regulate internal processes, including the immune system, to maintain homeostasis; however, organisms must also adapt to an everchanging external environment and adjust to variable environmental conditions (Goldstein, 2003). Stress elicits a deviation from an organism’s homeostatic setpoint, instigating a compensatory response to restore the internal steady state. An organism’s resilience within a changing external environment is dependent upon its ability to maintain or restore a homeostatic steady state through stress challenges regardless of their type (social, physiological, environmental, or pathogenic). Translating this concept to animal agricultural production, the ability to maximize the genetic potential of livestock and poultry is dependent upon the animals’ ability to appropriately respond to stressors such that internal homeostasis is maintained. When contemplated this way, immune system activation and the resulting inflammation can be thought of as a means to restore the host’s steady state when its physiological homeostasis is challenged instead of an undesired detriment to productivity.


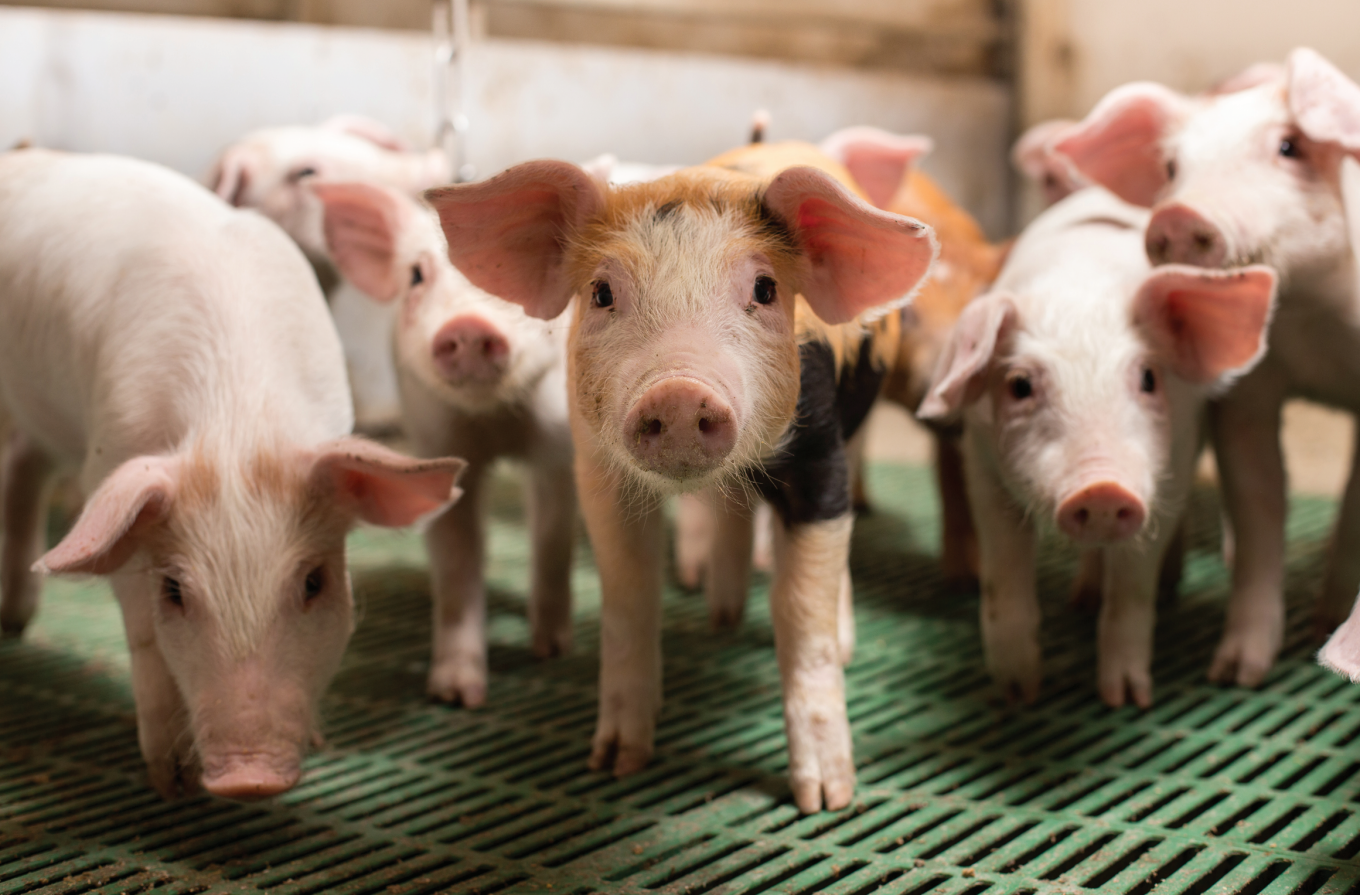


## Inflammation and Animal Productivity

The immune system evolved along with metabolic processes to ensure an organism could grow, propagate, and survive. The life history theory in evolutionary science demonstrates that organisms optimize limited resources for reproductive success and undergo metabolic reprogramming to balance resource allocation between biological processes needed for success with those necessary for survival (Wang et al., 2019). For example, when there is an abundance of nutrients available in the environment, biological processes are shunted toward resource-consuming growth and reproduction whereas in unfavorable conditions such as nutrient scarcity or pathogenic challenges, resources are diverted to resource-sparing processes for survival. Immune activation and inflammation in response to a pathogenic challenge exemplifies the metabolic reprogramming that occurs to support the immune response needed for survival at the expense of growth and productivity. Inflammation associated with immune activation is brought about by a cascade of proinflammatory cytokines including interleukin (IL)-1, IL-6, and tumor necrosis factor (TNF)-α, and these cytokines signal through the neuroendocrine pathway to influence feed intake and how dietary nutrients will be partitioned in the animal.

Inflammation can be considered the cost of productivity in animal agriculture and is exemplary of the interplay between the immune system and metabolism. Furthermore, it represents a mitigation point for managing the nutrient resources between growth and survival. Damping immune activation and inflammation provides an obvious enhancement in growth and productivity in livestock and poultry but that must be balanced with the role inflammatory processes have in immune development and healing of damaged tissue. For instance, inflammation is a critical signal for immune development in the young animal. Immune system development, in coordination with early microbial establishment in the gastrointestinal tract is necessary to appropriately educate immune system responses to the external environment. In addition, the inflammatory process associated with the arachidonic acid pathway is critical for the tissue healing that is important for recovery of the damaged gastrointestinal epithelium resulting from weaning the young pig from milk to a grain-based diet or for recovery of the reproductive tract in the cow after parturition. Inflammation associated with challenges from the external environment or with tissue recovery is tightly regulated and is resolved through the upregulation of anti-inflammatory cytokines such as IL-10 to prevent an overexuberant immune response that would be counteractive to the animal’s survival (Elsasser et al., 2008). When pro- and anti-inflammatory responses are regulated appropriately, resource allocation is balanced to support the animal’s metabolic processes for survival while optimizing growth and productivity.

## Conclusion

Immune responses evolved in animals to support their adaptability and survival within a continuously changing environment. A better understanding is needed in the field of animal science related to how the environment and early life exposures influence immunometabolism and metabolic programming throughout an animal’s productive life. Animals can then be managed to support the development of appropriate immunological processes and allocation of nutrient resources to ensure survival while also optimizing growth and reproduction.

## About the Author

Dr. Ellen is the Swine Technical Service Manager at Arm & Hammer Animal and Food Production where she provides technical sales support for probiotic and yeast-based feed additives. She received her B.S. in animal science from North Carolina State University, M.S. in animal nutrition from Oklahoma State University, and Ph.D. from the University of Arkansas where her research focused on nutritional immunology of the young pig. Her career in the animal agriculture industry began in Research & Development at Agtech Products, Inc. where she worked on the discovery of novel probiotic bacteria for swine, poultry, and ruminant applications. A series of company acquisitions resulted in Ellen continuing her work in R&D with Danisco and Dupont. Ellen’s work in R&D has resulted in 18 peer-reviewed publications, 15 US patents, and over 50 international patents on microbial products for swine, poultry, and ruminant applications. She has been an active member of ASAS since 1996. **Corresponding author:**ellen.davis@churchdwight.com
